# The effect of local land use on aerial insectivorous bats (Chiroptera) within the two dominating crop types in the Northern-Caribbean lowlands of Costa Rica

**DOI:** 10.1371/journal.pone.0210364

**Published:** 2019-01-15

**Authors:** Priscilla Alpízar, Bernal Rodríguez-Herrera, Kirsten Jung

**Affiliations:** 1 Escuela de Biología, Universidad de Costa Rica, San Pedro de Montes de Oca, San José, Costa Rica; 2 Institute of Evolutionary Ecology and Conservation Genomics, University of Ulm, Ulm, Baden-Württemberg, Germany; University of Reunion Island, RÉUNION

## Abstract

Land transformation into agricultural areas and the intensification of management practices represent two of the most devastating threats to biodiversity worldwide. Within this study, we investigated the effect of intensively managed agroecosystems on bat activity and species composition within two focal areas differing in landscape structure. We sampled bats via acoustic monitoring and insects with flight interception traps in banana and pineapple monoculture plantations and two nearby protected forested areas within the area of Sarapiquí, Costa Rica. Our results revealed that general occurrence and feeding activity of bats was higher above plantations compared to forested areas. We also recorded higher species richness at recording sites in plantations. This trend was especially strong within a fragmented landscape, with only four species recorded in forests, but 12 above pineapple plantations. Several bat species, however, occurred only once or twice above plantations, and forest specialist species such as *Centronycteris centralis*, *Myotis riparius* and *Pteronotus mesoamericanus* were only recorded at forest sites. Our results indicated, that mostly mobile open space and edge foraging bat species can use plantations as potential foraging habitat and might even take advantage of temporal insect outbreaks. However, forests are vital refugia for several species, including slower flying forest specialists, and thus a prerequisite to safeguard bat diversity within agricultural dominated landscapes.

## Introduction

Land use intensification and the increasing transformation of natural habitats into agricultural land are known as the greatest threats to biodiversity worldwide [1–4]. Today, agricultural areas cover approximately 40% of our planet’s terrestrial surface [5], a number which is expected to increase steadily [6] due to human population growth and resultant resource needs [7]. This trend is especially pronounced in tropical areas and lower income countries. Meanwhile those countries often harbor the most pristine and species-rich areas with high rates of endemism [8]. This raises the responsibility for the conservation of natural areas and emphasizes the need to include agricultural areas into biodiversity related studies [9].

In the Neotropics, a range of countries increasingly become aware of their responsibility for conservation and the potential of their natural diverse flora and fauna for green tourism. Among these countries, Costa Rica may be one of the good examples, as it has one of the best systems of protected areas in Latin America, with 1,861,715 ha, thus 26% of the country’s territory [10]. These areas are vital refugia for many species and help to safeguard the national biodiversity of Costa Rica [11]. Nevertheless, even Costa Rica experienced a significant transformation of forested areas into agricultural land in the last years. In 2011, 10% of the land was used for crop cultivation, an increase of 59,230 ha since 2008 (from 441,697 to 500,927 ha) [12]. Hereby pineapple and banana are the two fresh fruit crops with the most extensive areas in the country [12] and the largest gross value mostly cultivated for overseas export and covering nearly a fifth of the country’s export value per year (over 715,000 US dollars; 17% of agricultural exports each) [12]. Thus, besides sugar cane and coffee, the export of banana and pineapple safeguards the economy of Costa Rica and exemplifies the interconnection of the two most pressing concerns in tropical regions of the world: economic stability and the reduction of deforestation to mitigate climate change and biodiversity loss [7]. Consequently, one of the major challenges for any conservation strategy appears to be a profound understanding to what extent managed agricultural areas can still hold the original biota [13] and whether protected areas near agricultural land may mitigate the loss of biodiversity at the landscape scale.

Within the Neotropics about half of the mammalian fauna is represented by bats [14]. Due to their flight ability, they function as mobile links within the landscape [15] by providing crucial ecosystem services [16] such as pollination of native plants and commercial crops, regeneration of forest by seed dispersal and control of insect abundances by herbivore arthropod predation [17–18]. Bats are thus natural agents maintaining the ecological equilibrium within agriculturally dominated areas and thus important for sustainable land-use in the Neotropics. However, their ability to provide such crucial ecosystem services also depends on the structural composition of the landscape which determines accessibility [19–20], potential habitat [21] and resource diversity at larger scales [22].

Most studies focusing on the effect of agricultural land use on bats at the local scale have consistently demonstrated a decrease in species richness and a change in species assemblages [9] with increasing land use. Hereby the type of agricultural land management greatly influences the negative effects on biota [23]. While agroforestry systems can provide suitable habitat for a range of the naturally occurring species [24], monocultures are typically characterized by lower abundances, higher dominance of single species [25] and altered species assemblages. Differences in the potential of individual bat species to use an agricultural landscape have previously been linked to functional groups [19, 26], mobility and the degree of species specialization [27–28]. Forest specialists, such as narrow space foragers are less mobile and rarely occur within changed landscapes, while edge and open space foragers are rather mobile and are known to readily take advantage of fluctuating insect abundances, even in agricultural areas [29].

Within the current study we investigated the effect of local land use on aerial insectivorous bat species activity and species composition within the two dominating crop types in Costa Rica, banana and pineapple both planted in mono-cultures, in comparison to nearby protected forests. We worked in two different focal areas differing in landscape composition: a heterogeneous and fragmented landscape, which consisted of a relatively small forest fragment and small fields of organic pineapple plantations, and a homogeneous less fragmented landscape, represented by a large forested area with extensive conventional banana monocultures nearby.

We expected that species richness and abundance of bats decreased in banana and pineapple plantations compared to the respective forested areas. Due to landscape composition and loss in connectivity, we further expected a higher contrast in species richness and abundance patters between plantation and natural forests within the less fragmented focal area, characterized by large monocultures of bananas and the extensive forest area of La Selva Biological Station, which is known to provide a high biotic diversity. In comparison, we expected a smaller contrast in species richness and bat abundance between forest and pineapple plantations within the heterogeneous focal area as several landscape elements and remaining vegetation structures, could potentially maintain site accessibility for several bat species.

## Materials and methods

### Ethical statement

This study was conducted under the Resolution No. 056-2012-SINAC emitted by the Sistema Nacional de Áreas de Conservación (SINAC) in Costa Rica. Bat occurrence was assessed using passive acoustic monitoring, which is a non-invasive method. All monitoring sites were privately owned and were entered only with the agreement of their owners or managers. No protected species were sampled during this study.

### Study area

Our study was conducted from March to August of 2012 in two forest reserves and nearby pineapple and banana plantations of the Caribbean lowlands (0–400 meters above sea level) in Sarapiquí, of the province Heredia, Costa Rica. This area is the main region for banana and pineapple production in Costa Rica contributing to a large proportion of the fruit production value of the whole country [5]. The whole region is characterized by Caribbean tropical weather conditions, with a dry season from March to May and a rainy season from May to February. The mean annual temperature is 26°C and the mean annual precipitation around 3710 mm [30].

**Fig 1:**
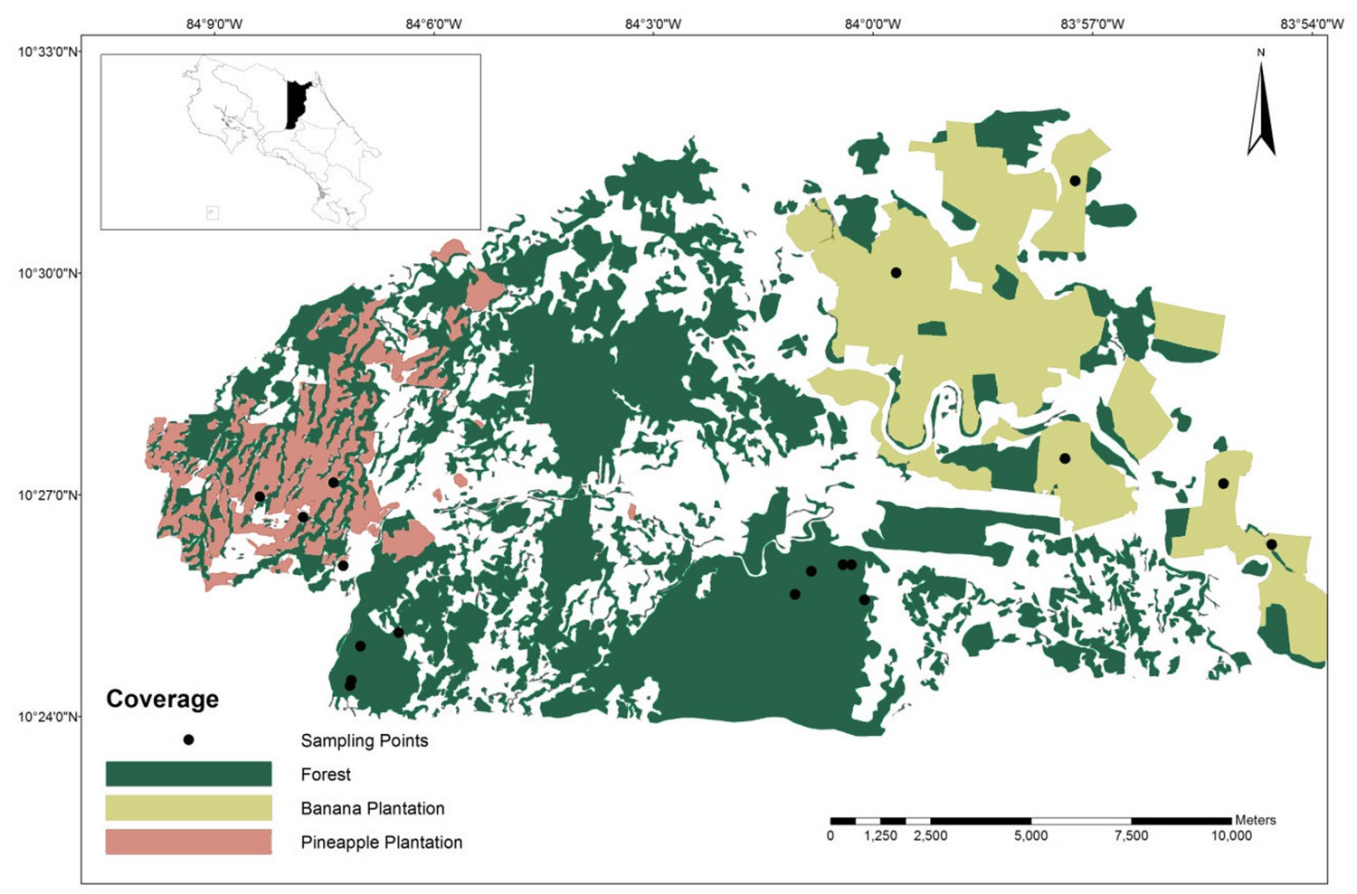
Map of the study area and the acoustic monitoring sites for bats in forest and plantation habitats, within Sarapiquí, Costa Rica. Image sources: Mosaic generated in SIG OTS from aerial photographies issued by the Costa Rica Airborne Research and Technology Applications (CARTA 2005), Centro Nacional de Alta Tecnología (CENAT) and Nasa Johnson Space Center (2014). Elaborated by M. Runnebaum.

Acoustic monitoring of bats and insect sampling was conducted at 18 sites within two focal areas: the forest of Tirimbina Biological Reserve and nearby pineapple plantations (fragmented area) and the forest of La Selva Biological Station including the surrounding banana plantations (homogenously structured area; Fig 1). Both focal areas are located between 40 and 200 meters of elevation and possess similar climatic conditions.

Tirimbina Biological Reserve covers an area of 412 ha which is mainly composed by primary forest with patches of secondary forest and early regeneration stages. Close to Tirimbina we used pineapple plantations of Collin Street Bakery, which are part of an organic farming project called Finca Corsicana, in La Virgen. Pineapple fields, with an average extent of 2–4 ha, were boarded by small creeks, gallery forest and near (200 m) small forest patches.

La Selva Biological Station (Organization of Tropical Studies, OTS), covers 1,600 ha of low land wet tropical primary and secondary forest. La Selva is bordered by Braulio Carrillo National Park, which contains more than 46,000 ha of forest land and is the core conservation unit of the 91,000 ha Cordillera Volcánica Central Biosphere Reserve [31]. Our recording sites in banana plantations near La Selva, were in the Nogal Nature and Community Project which is a designated area for sustainable land use and maintenance of high biodiversity. Banana plantations, with an average size of 200 ha are much larger than pineapple plantations, creeks and remnants of forest fragments are lacking and the landscape appears as a typical monoculture.

### Acoustic monitoring

Acoustic monitoring within these focal areas was conducted in a paired sampling design always simultaneously monitoring a forest and a plantation habitat site. Four monitoring sites in Tirimbina forest where thus paired with four sites in pineapple plantations, while five forest sites in La Selva where paired with five sites in banana plantations. Within each habitat, sites were at least 200 meters apart from each other and 100 meters away from habitat edges, roads or water bodies. Sampling points in the forests were located within secondary and primary forest. Recording devices were fixed at tree trunks and the microphone was directed towards openings of the forest understory to assure the recording of edge and potentially even open space foraging bats. Sampling always alternated between the two focal areas and each focal area was resampled in an interval of about 15–21 days. Sampling was not conducted under full moon conditions and was rescheduled to another night in case of rain fall.

Recordings where taken with an automatic acoustic ultrasound device (Song Meter SM2 Bat recorders, Wildlife acoustics) at a sample rate of 192 kHz and 16 bit. SongMeters were programmed to continuous recording and nightly monitoring sessions started at 17:30 until midnight. At each site monitoring continued for three successive nights.

### Recording analysis

Acoustic analysis was limited to the first three and a half hours (17:30–21:00 h) of each recording night. This recording period covers the major peak in bat activity during the night [32] and we thus are confident that our analysis covers a representative sample of the bat assemblage and activity of a given site. We first used an automatic detection system for bat calls within each recording night [33–34]. Individual sound sequences where then further analyzed for species identification using the software Avisoft Saslab Pro 5.1.20 (Raimund Specht, Avisoft Bioacustics, Berlin, Germany). Hereby we used a spectrogram with an FFT of 1024, a Hamming window and an overlap of 96%.

To assess bat species activity at a site, as an indicator for the relative intensity of habitat use, we counted the number of passes over our microphone. Hereby, a pass was defined as a minimum of three consecutive echolocation calls. Two passes were separated by a time interval exceeding three times the regular pulse interval of the respective species [32]. In addition, we assessed foraging activity by evaluating the number of feeding buzzes per night and site. Feeding buzzes are characterized by call sequences emitted at a high repetition rate just before prey capture attempts [35].

We manually identified sound sequences to species based on a reference call library from E. Kalko and K. Jung at the Ulm University, Germany, and by consulting existing literature on echolocation calls [28, 36–40]. Due to the sampling rate of the recorder device, we could not record the Proboscis Bat (*Rhynchonycteris naso*, Emballonuridae) known to frequently occur in the study area. In addition, acoustic identification of some open space foraging bats, especially in the genera of *Eumops* and *Molossus* is very difficult [37]. We thus grouped all recordings of *Eumops* into *Eumops spec*. and regarded *Molossus currentium and M*. *sinaloae*, which overlap substantially in call structure and frequencies, as an acoustic species complex.

### Insect sampling

Parallel to the acoustic survey of bats we sampled insects to estimate potential prey availability. Insects where trapped using passive flight interception traps [41] which were installed during the same nights and in the same locations as the acoustic recording devices at a height of three meters. We conserved the samples obtained from the flight interception traps in 70% alcohol and identified the insects to the lowest taxonomic level possible, and thus mostly to family level. Due to the low sample size of captured insects, samples of the three successive monitoring nights per sites where combined for further analysis. Insects were dried in an oven at 70°C for 24 hours their dry weight was determined using an electronic micro balance (Cahn C-33, precision 0.001 mg). In further analysis, dry weight was considered as an index of resource abundance. To estimate differences in insect diversity between habitats we calculated the effective number of insect families [42].

### Data analysis

We estimated inventory completeness using sample-based species accumulation curves with 1000 randomizations (Estimate S) [43]. Expected number of species were estimated based ICE species richness estimator (S_est_), which we derived using the estimator choice framework by [44]. Percentage of inventory completeness was then calculated by dividing the number of observed species in each site (S_obs_) by estimated number of species (S_est_) and multiplied by 100.

To compare rarefied species richness and dominance of single species per habitat, we used rarefaction analysis with 1000 iterations and independent sampling. Dominance was assessed using the Berger-Parker Index. These calculations were based on presence absence data of bat species per night and site to avoid an overestimation of species abundance due to repeated sampling of individuals passing our microphone during the same sampling night [28]. These analyses were conducted in Ecosim [43].

Following analyses were conducted using R 2.15.3 (R Development Core Team 2013). Poisson distributed generalized linear mixed effect models (glmm, library lme4) [45] were used to assess if bat activity differs between habitats. We standardized bat activity by time and calculated the number of bat passes per recording hour [26]. Passes per hour were included as dependent variable and recording sites and sampling nights were included as random factors due to repeated sampling. Generalized linear mixed effect models were also used to assess the differences in resource abundance (dry weight of insects) and resource diversity (effective number of families) between habitats, here we only included site as random factor, as data of the three successive nights had been pooled.

Finally, we assessed the effect of habitat, resource abundance and resource diversity on the feeding activity of bats using a glmer (package: lme4) [45], sites and nights were included as random factors into the model. Significant responses in models were evaluated by conducting multiple comparison tests using Tukey contrasts implemented in the ‘multcomp’ package in ‘R’ [46].

To explore the differences in species composition between the two focal areas and each site category (plantation and forest) we conducted a nonmetric multidimensional scaling (NMDS) based on Bray-Curtis dissimilarities. For the NMDS we used mean activity of a species at a site during the three sampling nights. As NMDS is very sensitive to rare species, thus we excluded all species with less than 10 passes over the whole study period from the dataset. To test for differences in species composition between focal areas, habitat types and site categories we used a permutative multivariate analysis based on distance matrices (adonis, package vegan) [47]. In addition, we investigated differences in the variance of species composition between focal areas, habitat types and site categories using a multivariate test of group variance (betadisper, package vegan) [47].

## Results

**Fig 2:**
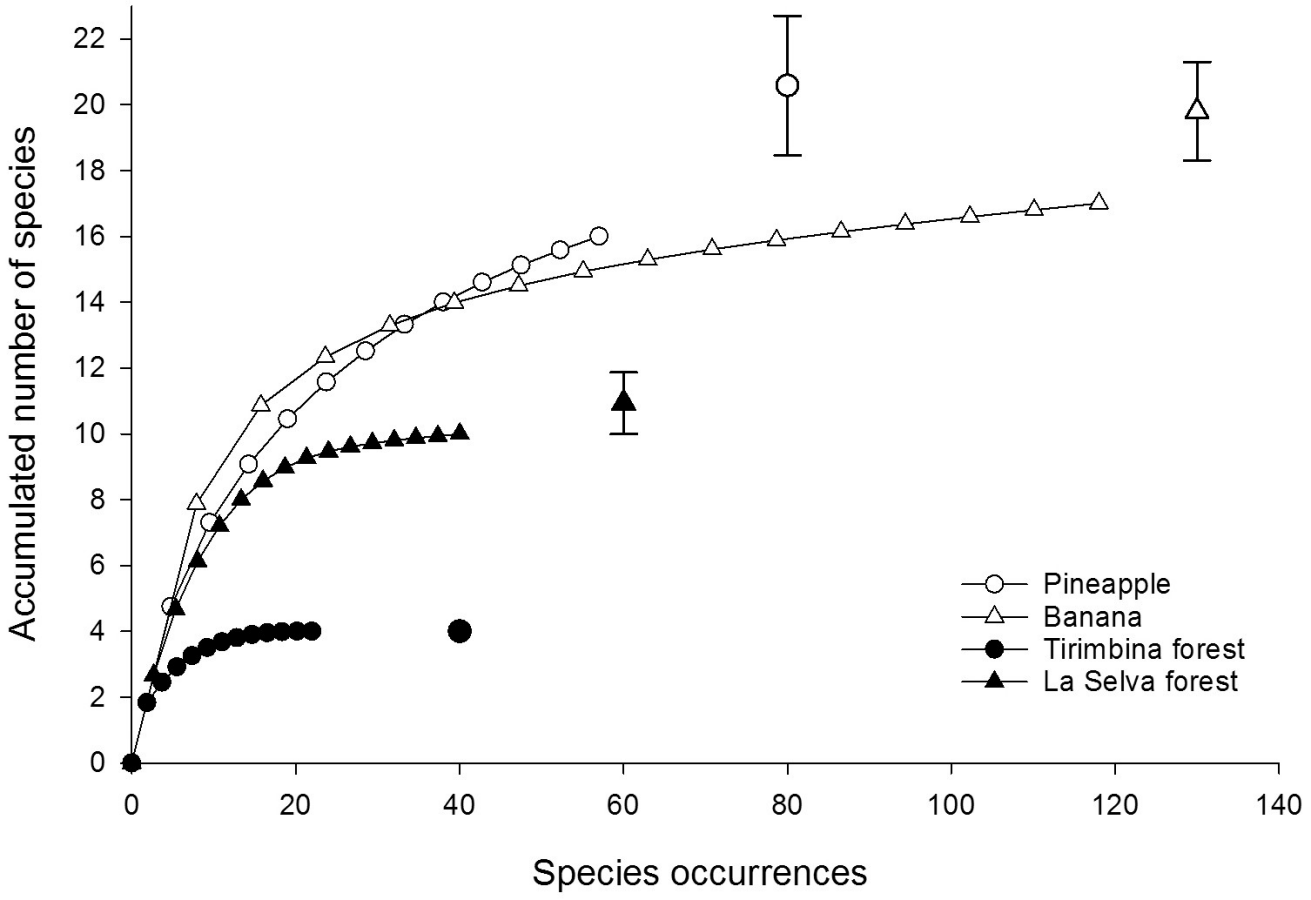
Accumulated number of species occurrences in plantation and forest habitat based on acoustic monitoring within the two focal areas in Sarapiquí, Costa Rica. Separate points stand for the expected number of species (with standard deviation) calculated using the ICE species richness estimator.

In total, we obtained 4,225 bat passes corresponding to a total of 220 occurrences of 21 different aerial insectivorous bat species over the whole sampling period (Table 1). At all site categories species richness steadily increased with the number of sampling nights and reached an inventory completeness of 76–100%, indicating that our sampling was sufficient to compare species assemblages between habitats in further analysis (Fig 2).

**Table 1:** Bat activity (total number of passes) and feeding activity (total count of feeding buzzes) of aerial insectivorous bats in each site category by species.

Species	Abbr.	Family^a^	Number of passes				Total count of feeding buzzes
			Banana	La Selva	Pineapple	Tirimbina	
*Centronycteris centralis*	*C*.*cen*	EMB	0	629	0	574	18
*Cormura brevirostris*	*C*.*bre*	EMB	16	19	3	0	1
*Diclidurus albus*	*D*.*alb*	EMB	1	0	0	0	0
*Eptesicus brasiliensis*	*E*.*bra*	VES	81	0	20	0	11
*Eptesicus furinalis*	*E*.*fur*	VES	9	0	2	0	2
*Eumops* sp.	*Eum*	MOL	98	5	82	0	0
*Lasiurus ega*	*L*.*ega*	VES	9	0	1	0	0
*Molossus currentium/sinaloae*	*M*.*cs*	MOL	992	80	260	0	94
*Molossus molossus*	*M*.*mol*	MOL	5	0	7	0	0
*Myotis albescens*	*M*.*alb*	VES	42	0	10	0	1
*Myotis elegans*	*M*.*ele*	VES	7	0	0	0	1
*Myotis nigricans*	*M*.*nig*	VES	316	31	152	0	26
*Myotis riparius*	*M*.*rip*	VES	1	9	0	0	0
*Noctilio* spp.	*Noct*	NOC	15	0	1	0	0
*Peropteryx kappleri*	*P*.*kap*	EMB	44	1	3	0	2
*Peropteryx macrotis*	*P*.*mac*	EMB	4	0	2	0	1
*Pteronotus davyi*	*P*.*dav*	MOR	0	0	1	0	0
*Pteronotus gymnonotus*	*P*.*gym*	MOR	1	0	0	0	0
*Pteronotus mesoamericanus*	*P*.*mes*	MOR	0	20	2	4	1
*Rhogeessa io*	*R*.*io*	VES	0	0	1	0	0
*Saccopteryx bilineata*	*S*.*bil*	EMB	394	90	12	6	0
*Saccopteryx leptura*	*S*.*lep*	EMB	136	25	13	2	20
Not identified			227	70	55	1	2

^a^ Bat families recorded in this study were: Emballonuridae (EMB), Molossidae (MOL), Mormoopidae (MOR), Noctilionidae (NOC), and Vespertilionidae (VES)

**Fig 3:**
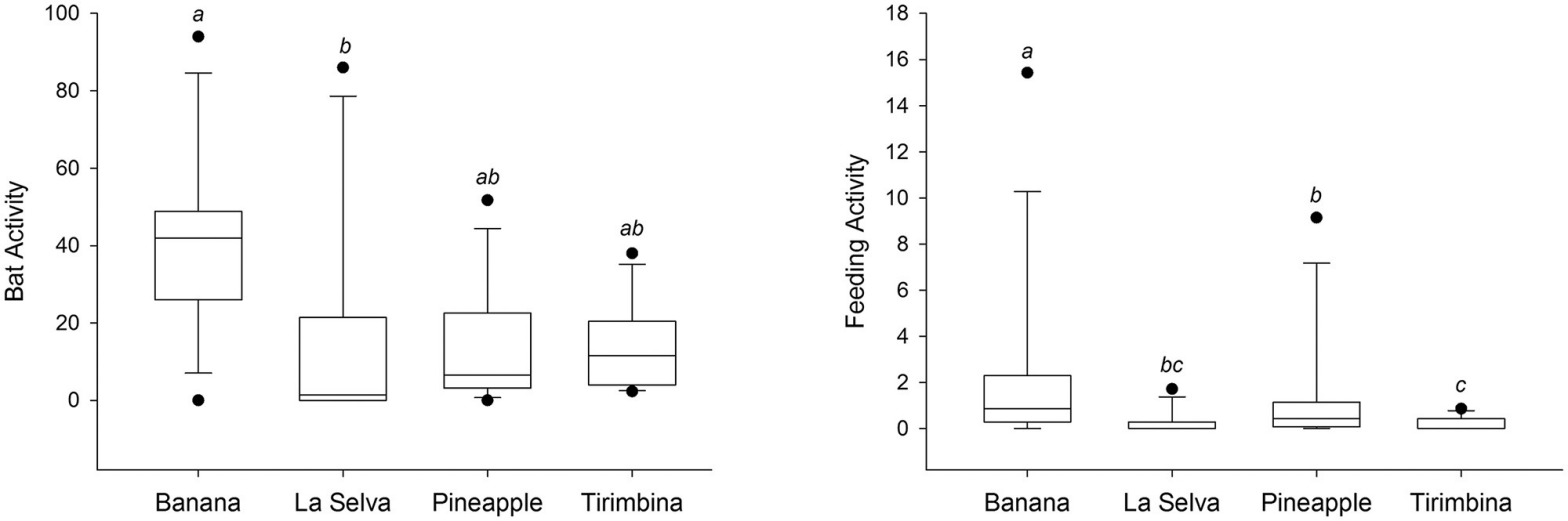
Differences in bat activity and feeding activity between the four habitat categories in Sarapiquí, Costa Rica. (A) Bat activity (number of passes), significance value is p <0.05. (B) Feeding activity (number of feeding buzzes), significance values are p <0.05 for the banana-pineapple pair and p < 0.001 for the three remaining significant pairs. Bars represent standard deviation.

Bat occurrence, activity and feeding activity differed substantially between the two focal areas, all being substantially higher in the homogenous landscape of the focal area of La Selva and Banana plantations compared to the heterogeneous landscape. Banana plantations revealed the highest bat activity, although with a very high variation in activity levels between individual recording sites (Fig 3a; Chi-square = 6.77, p > 0.05).

In contrast to our hypothesis, bat occurrence and species richness was higher in plantations compared to forests (Table 2). This was a consistent result in both focal areas. In addition, both, observed species richness and rarefied species richness, in plantations exceeded species richness at forest sites.

**Table 2:** Activity and percentage of inventory completeness of aerial insectivorous bats for each habitat category.

Site category	OC	S(obs)	S(est)	Single OC’s	Complete-ness (%)	Rarefied Species richness	Dominance	Activity	Feeding
**Fragmented landscape**	114	17							
Tirimbina	22	4	4	0	100	4	0.55	586	7
Pineapple	92	19	21.5	3	76.0	12	0.18	571	56
**Homogenous landscape**	158	19							
La Selva	40	10	10.26	1	97.5	9	0.20	910	12
Banana	118	17	18.8	3	90.2	11	0.19	2158	125

Number of species occurrences (OC), observed species richness (*S*_*obs*_), estimated species richness (*S*_*exp*_) and percentage of inventory completeness for each site category within the two focal areas. Species richness and dominance are rarefied to 20 accumulated number of a species occurrences. Also listed are bat activity (passes) and feeding activity (capture attempt) of aerial insectivorous bats.

Differences in species richness were especially pronounced in the heterogeneous focal area between forest sites in Tirimbina and pineapple plantation sites. Within the heterogeneous focal areas rarefaction also indicated a more pronounced difference in species relative abundance, with a much higher dominance at the forest sites in Tirimbina (Table 2).

**Fig 4:**
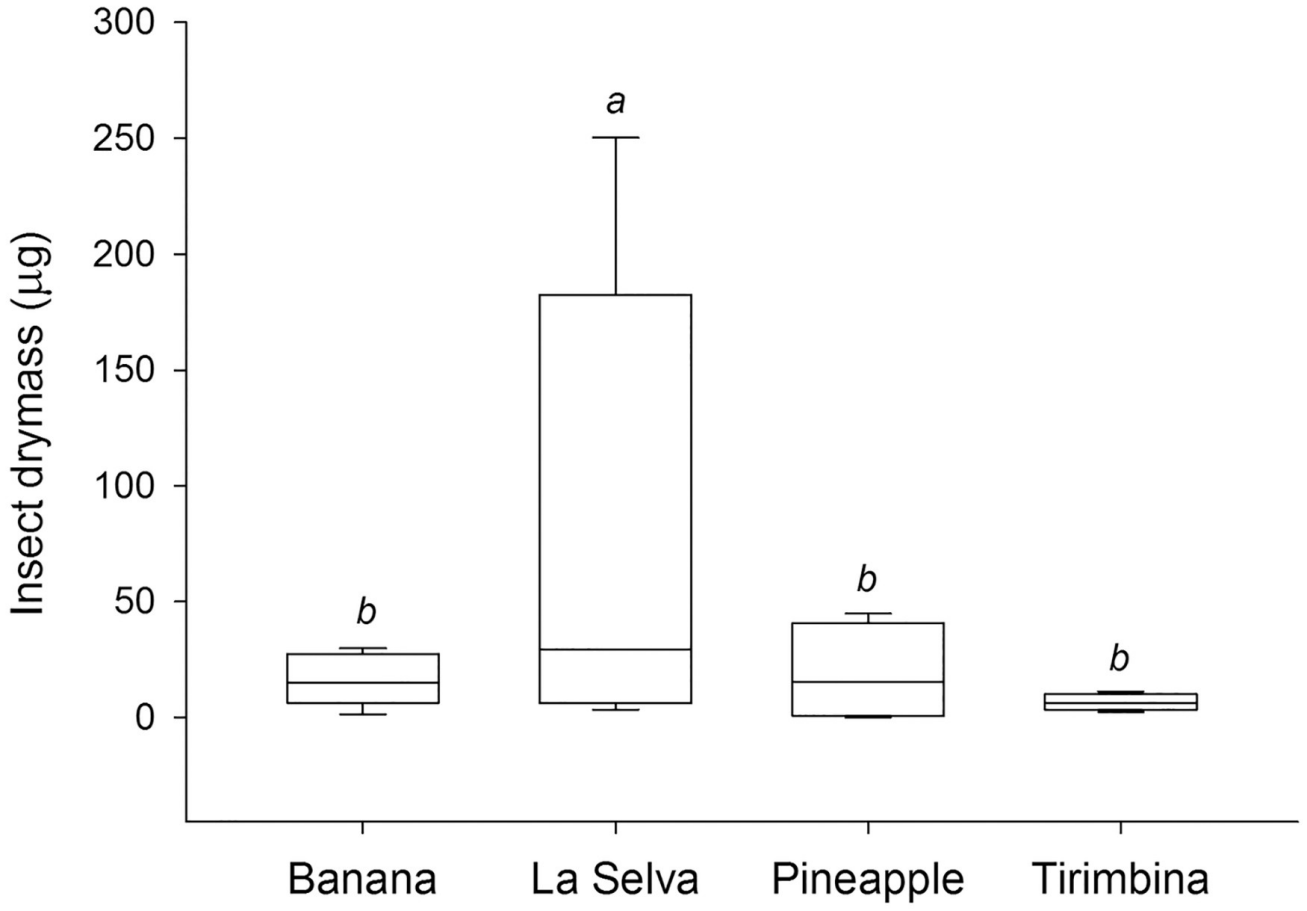
Differences in insect abundance (drymass) between the four habitats types. Bars represent standard deviation. Significance value is p < 0.001.

Meanwhile insect abundance at the forest of La Selva exceeded insect abundance at all other sites (Fig 4; Chi-square = 425.64, p < 0.001). In contrast, insect diversity did not differ significantly between habitat types, but the variation in insect diversity was generally larger at the heterogeneous focal area comprising Tirimbina forest and pineapple plantations.

In both focal areas feeding activity in plantations exceeded the feeding activity in the respective forest habitat (Fig 3b). In addition, feeding activity increased only slightly with resource abundances (p < 0.05) but highly significant with an increase in the diversity of insect families (p < 0.001, Table 3).

**Table 3:** Results of the generalized linear mixed effect models for bat activity and feeding activity.

	Parameters	Estimate	SE	Z	P -values	
**Bat activity**	*Intercept*	*3*.*49*	*0*.*58*	*5*.*99*	*6.5 e^-14^*	***
deviance: 436.4	La Selva	-2.16	0.85	*-2*.*54*	*0.009*	**
	Pineapple	-1.44	0.88	-1.64	*0*.*13*	*n*.*s*.
	Tirimbina	-1.22	0.87	*-1*.*40*	*0*.*20*	*n*.*s*.
**Feeding activity**	*Intercept*	*0*.*42*	*0*.*66*	*0*.*64*	*n*.*s*.	
deviance: 225.3	La Selva	-2.86	0.73	-3.91	*9.1 e^-05^*	***
	Pineapple	-1.43	0.60	-2.38	*0.018*	*
	Tirimbina	-4.66	1.02	-*4.54*	*5.6e^-06^*	***
	Resource abundance	-0.04	0.02	-*1.99*	*0.046*	*
	Resource diversity	-0.30	0.09	*3.15*	*0.002*	***

“***” indicates p values <0.001,

“**”p values <0.01,

“*”p values <0.05.

“n.s.” indicates non-significant results.

**Fig 5:**
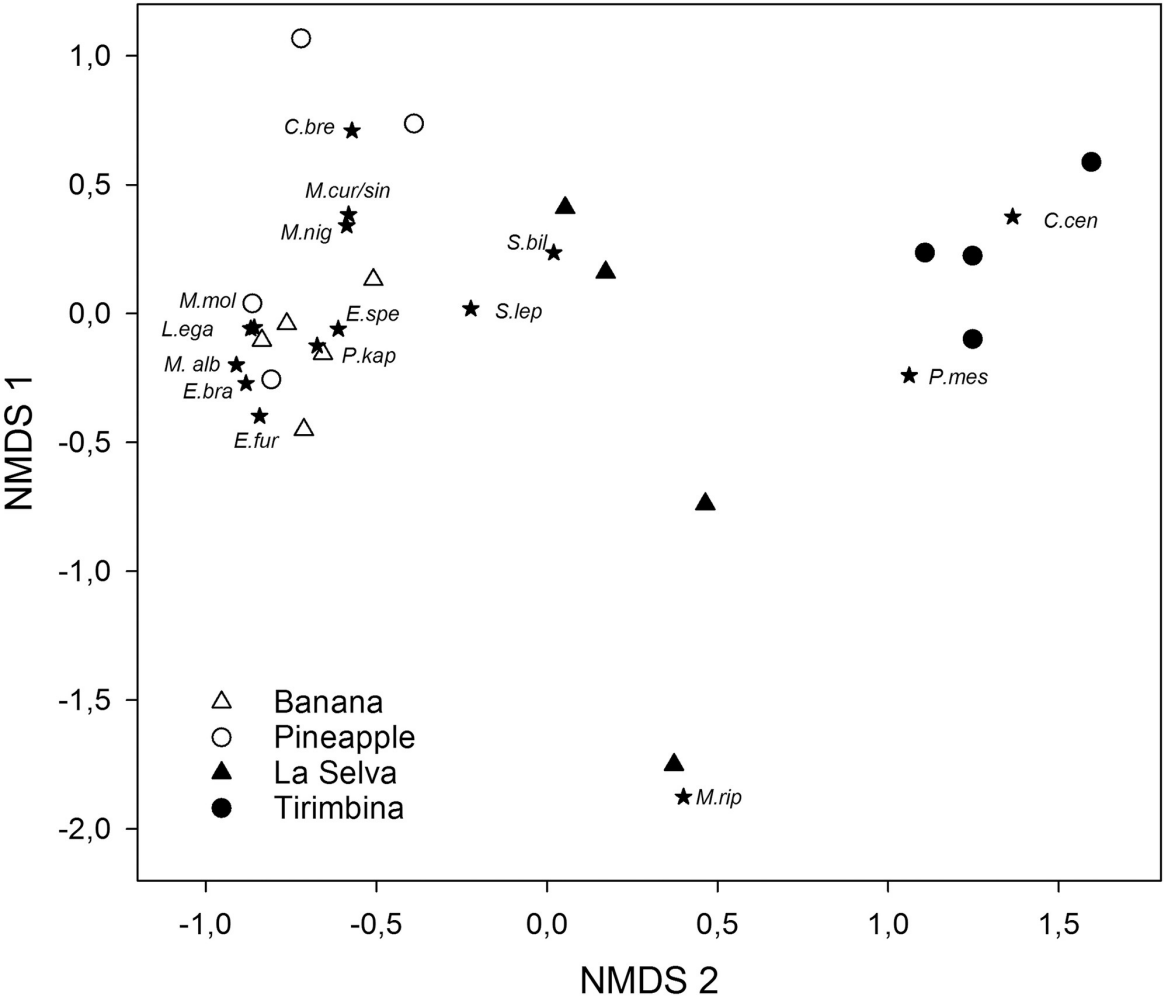
NMDS of recording sites. Non-metric multidimensional scaling of recording sites based on Bray-Curtis dissimilarities species composition of aerial insectivorous bats weighted by their mean activity at each recording site in Sarapiquí, Costa Rica.

Non-metric multidimensional scaling (final stress = 0.0880, linear fit of ordination distance and observed dissimilarity R^2^ = 0.966) separated recordings sites based on differences in species composition. Recording sites in plantations hereby grouped close together and clearly separated from recording sites of both forest areas (Fig 5). Permutative multivariate analysis of variance indicated that species composition at recording sites differed significantly between both focal areas (F _1,13_ = 2.27, p = 0.034), habitat types (F _1,13_ = 9.47 p< 0.001) and site categories (F _1,13_ = 2.86, p = 0.024). Variance in species composition differed significantly between forest and plantations sites within both focal areas (Tirimbina-pineapple: p <0.05, La Selva-banana: p<0.01), indicating a significantly higher variance between individual forest recording sites compared to plantations. In addition, variance in species composition at recording sites of La Selva exceeded the variance in Tirimbina (p<0.01).

## Discussion

The ubiquity of agricultural land use [9], the intensification of management strategies [4] accompanied by increasing loss of landscape heterogeneity, highlights the importance of including agricultural used land when aiming for biodiversity conservation at the landscape-level [48]. This is especially important in tropical areas where high levels of biodiversity and endemism directly interact with human economic ambitions.

Within the current study we investigated habitat use of insectivorous bats within an area of Costa Rica that is currently undergoing rapid habitat alteration from natural areas towards intensively managed banana and pineapple monocultures. We were interested in the contrasting habitat use and foraging activity of bat species between two protected forests and plantations. It has previously been shown, that structurally complex landscapes might be able to buffer the negative effects of intensified land use, enhancing local diversity in agroecosystems [49]. We thus contrasted habitat use of insectivorous bats between a homogenous (large traces of forest and banana monocultures) and a rather heterogeneous landscape (small forest fragment and small pineapple plantations).

In contrast to our expectations, bat species richness and foraging activity were higher in both plantation types compared to forests, and our data revealed highest activity and foraging activity above the large, extensive monocultures of banana plantations. Our data thus suggests that plantations can indeed function as a potential foraging ground for many bat species. Typical banana plants for export in Costa Rica are planted in large monocultures at a density of one plant per of about 3–5 m^2^. They grow quite fast and typically reach an altitude of about 3–5 m. Below the vegetation layer of the leaves remains a well-covered space that may provide shelter from potential aerial predators. In addition, the vegetation layers may provide habitat for a range of herbivore insects. Although our data did not reveal an increased insect dry mass in banana plantations compared to forest sites, bats might profit from fluctuating insect outbreaks within the plantations, which our limited insect sampling might have missed. This is likely, considering that banana monocultures heavily depend on high inputs of pesticides and insecticides to achieve and sustain high yields [50]. In contrast to banana plantations, pineapple plantations are poor in vegetational structure, however rich in insect abundance. Most of these small plantations were bordered by vegetation, which potentially serves as a connecting element to remaining and nearby forest patches [26, 29] and might assure their accessibility by bats. These plantations were also using organic farming methods, with hardly to none agrochemical input and natural fertilizers. It has been observed that these agricultural practices also contribute to higher insect abundance and diversity in agricultural landscapes [51].

We also found a higher species richness of bats above plantations compared to the nearby forested sites. We argue that this is partly due to an increased detection of opportunistic open and edge foraging aerial insectivores at plantation recording sites, which might have been missed at our forest sites. This is likely given by the high variability of species numbers between individual forest recording sites and might be due to the structural heterogeneity within both forest areas. In contrast to our expectations, differences in species richness were especially pronounced in the heterogeneous focal area between forest sites in Tirimbina and pineapple plantation sites. Rarefaction also indicated a much higher dominance of single species at the forest sites in Tirimbina (Table 2). We argue that this might be due to the logistic limitation of our sampling sites, as previous data shows that Tirimbina Biological Reserve has higher species richness of aerial insectivore bats than the one found in this study [52].

Our results further indicate an altered community structure in plantations with a higher number of rare and seldom occurring species in both plantation types. Species such as *Diclidurus albus* (Emballonuridae), *Pteronotus davyi* (Mormoopidae), and *Pteronotus gymnonotus* (Mormoopidae), occurred only once above either banana or pineapple plantations. These species could either be classified as forest dependent and/or cave dependent species [53] due to their roosting requirements [54–55] and are more prone to habitat changes. This is also in accordance with our results from the NMDS which showed, that mostly open and edge foraging species occurred above plantations sites. Those species are highly mobile and fast fliers along or above the vegetation. Several previous studies showed that species with such high mobility and large activity ranges are more likely to persist in modified landscapes [27, 56–58] and thus can take advantage of fluctuating insect accumulations above plantations. In contrast, we recorded *Centronycteris centralis* (Emballonuridae), *Pteronotus mesoamericanus* (Mormoopidae), and *Myotis riparius* (Vespertilionidae) primarily at forest sites. All three species are known to predominantly forage within rather dense vegetation of the forest understory or within small forest gaps. *Centronycteris centralis* has previously been classified as a forest specialist [32, 36, 59] and is particularly vulnerable to anthropogenic changes [28].

This underlines that forested areas are a prerequisite for species diversity at the landscape scale and emphasizes that protected forest areas such as La Selva and Tirimbina, within the increasing agricultural dominated region of Sarapiquí, are vital refugia for several bat species and thus help to safeguard the regional bat diversity.

## Conclusions

Costa Rica has one of the best systems of protected areas in Latin America; 26% of its land is under some sort of protection or conservation regime [10]. Nonetheless, this country has been—and still is—experiencing a significant transformation in land use from natural to agricultural lands [12]. This trend in land transformation is widely observed all over the Tropics, affecting biodiversity and ecosystem function. It is thus crucial to understand to what extent managed agricultural areas can still hold the original biota [13] and whether protected areas near agricultural land may mitigate the loss of biodiversity due to land-use.

Our results demonstrate that highly mobile and flexible animals, such as aerial insectivorous bats, can tolerate land use changes to some degree and use plantations as foraging grounds. Bat abundance and foraging activity was high above plantations indicating that mostly mobile and edge foraging bats species with rather high mobility can take advantage of insect accumulations and provide vital ecosystem service by controlling insect populations. Given the intense foraging activity of aerial insectivorous bats above agroecosystems we argue that pineapple and banana producers should be integrated into conservation management via educational programs, illustrating the ecological role of bats as insect controls, to assure the conservation of connective elements and forest fragments along and besides their plantations. This is important as forest specialist species foraging along or within the vegetation are highly vulnerable to habitat changes and depend on natural habitat conditions. To protect their persistence, the conservation of natural habitats is vital and most relevant. Finally protecting tbat habitat and encouraging producers to boost the presence of aerial insectivorous bats in their plantations, might decrease the high needs of pesticide use due to natural pest control and argument for organic agricultural practices in the country.

## Supporting information

S1 Table Total insect individuals captured in the flight interception trap by family in each site category. (DOCX)
