# Supplementary material for: The effect of local land use on aerial insectivorous bats (Chiroptera) within the two dominating crop types in the Northern-Caribbean lowlands of Costa Rica
Source: PLoS One. 2019 Jan 15;14(1):e0210364. doi: 10.1371/journal.pone.0210364 (PMC6333354; doi:10.1371/journal.pone.0210364)
Supplement: S1 Table — (DOCX) [file pone.0210364.s001.docx]

**S1 Table. Total insect individuals captured in the flight interception trap by family in each site category.**

| **Order** | **Family** | **Site category** | | | |
| --- | --- | --- | --- | --- | --- |
|  |  | **Banana monoculture** | **La Selva forest** | **Pineapple monoculture** | **Tirimbina forest** |
| Aphidoidea | Aphididae | 6 | 0 | 0 | 0 |
| Coleoptera | Anthicidae | 0 | 0 | 0 | 1 |
|  | Bostrichidae | 0 | 0 | 1 | 0 |
|  | Carabidae | 0 | 0 | 1 | 2 |
|  | Chrysomelidae | 0 | 0 | 2 | 2 |
|  | Ciidae | 0 | 0 | 1 | 0 |
|  | Coccinelidae | 1 | 2 | 2 | 1 |
|  | Curculionidae | 2 | 2 | 8 | 7 |
|  | Elateridae | 0 | 1 | 0 | 0 |
|  | Erotylidae | 1 | 0 | 2 | 1 |
|  | Eucnemidae | 0 | 2 | 0 | 0 |
|  | Leiodidae | 0 | 1 | 0 | 0 |
|  | Nitidulidae | 0 | 1 | 2 | 0 |
|  | Ptiliidae | 0 | 2 | 0 | 0 |
|  | Ptylodactylidae | 1 | 0 | 0 | 0 |
|  | Scarabaeidae | 0 | 1 | 5 | 1 |
|  | Staphilinidae | 4 | 3 | 104 | 1 |
|  | Tenebrionidae | 0 | 0 | 2 | 1 |
| Collembola | Not identified | 1 | 1 | 0 | 0 |
| Diptera | Acroceridae | 0 | 0 | 2 | 0 |
|  | Cecidomyiidae | 0 | 3 | 0 | 0 |
|  | Ceratopogonidae | 5 | 0 | 3 | 0 |
|  | Chironomidae | 1 | 0 | 0 | 0 |
|  | Chloropidae | 1 | 0 | 2 | 0 |
|  | Culicidae | 0 | 1 | 2 | 0 |
|  | Drosophilidae | 6 | 1 | 3 | 0 |
|  | Psychodidae | 55 | 0 | 0 | 0 |
|  | Sarcophagidae | 1 | 0 | 0 | 0 |
|  | Scatopsidae | 0 | 0 | 2 | 0 |
|  | Simuliidae | 10 | 1 | 1 | 0 |
|  | Tephritidae | 0 | 0 | 2 | 0 |
|  | Ulidiidae | 1 | 0 | 0 | 0 |
|  | Xelomyidae | 0 | 0 | 1 | 0 |
| Hemiptera | Achilidae | 0 | 1 | 0 | 1 |
|  | Alydidae | 0 | 0 | 1 | 1 |
|  | Cercopidae | 1 | 0 | 0 | 1 |
|  | Cicadellidae | 0 | 1 | 2 | 0 |
|  | Coreidae | 0 | 0 | 1 | 1 |
|  | Cydnidae | 0 | 2 | 1 | 3 |
|  | Fulgoridae | 0 | 0 | 1 | 0 |
|  | Miridae | 2 | 0 | 0 | 0 |
|  | Pentatomydae | 0 | 1 | 0 | 0 |
|  | Pyrrhocoridae | 1 | 0 | 0 | 0 |
|  | Scelionidae | 1 | 0 | 0 | 0 |
| Hymenoptera | Apidae | 0 | 0 | 0 | 1 |
|  | Bethylidae | 0 | 1 | 0 | 0 |
|  | Formicidae | 8 | 13 | 5 | 7 |
|  | Pergidae | 0 | 1 | 0 | 0 |
|  | Trigonalidae | 0 | 1 | 0 | 1 |
|  | Vespidae | 0 | 0 | 0 | 1 |
| Lepidoptera | Arctiidae | 0 | 0 | 1 | 0 |
|  | Galachioidea | 0 | 0 | 1 | 0 |
|  | Non identified Microlepidoptera | 1 | 1 | 0 | 0 |
| Orthoptera | Gryllidae | 0 | 1 | 0 | 0 |
|  | Tettigonidae | 0 | 0 | 0 | 1 |
| Protura | Protura | 0 | 2 | 0 | 0 |
| Psocoptera | Not identified | 0 | 0 | 15 | 0 |
| Thysanoptera | Anisolabidae | 0 | 0 | 0 | 2 |
|  | Labiidae | 0 | 0 | 0 | 1 |
